# Dapagliflozin effects on right ventricular function, pulmonary pressure and coupling: a propensity score analysis from the RIVED-HF

**DOI:** 10.1093/eschf/xvag230

**Published:** 2026-09-01

**Authors:** Alberto Palazzuoli, Gaetano Ruocco, Sara Franceschi, Giuseppe Schirò, Alessia Petrini, Niccolò Manetti, Andrea Salzano, Paolo Severino, Marco Guazzi, Giulia Crisci, Francesco Barillà, Enrica Mariano, Frank L Dini, Mariafrancesca Di Santo, Pasquale Perrone Filardi, Giovanni Sorrentino, Stefano Ghio, Giuseppe M C Rosano

**Affiliations:** Cardiovascular Diseases Unit, Le Scotte Hospital University of Siena, Italy; National Institute for Cardiovascular Research, Bologna, Italy; Veris Delli Ponti Hospital, ASL Lecce, Scorrano, Lecce, Italy; Department of Economics and Statistics, University of Siena, Siena, Italy; Cardiovascular Diseases Unit, Le Scotte Hospital University of Siena, Italy; Cardiovascular Diseases Unit, Le Scotte Hospital University of Siena, Italy; Cardiovascular Diseases Unit, Le Scotte Hospital University of Siena, Italy; Department of Translational Medical Sciences, ‘Federico II’ University, Naples, Italy; Interdepartmental Center for Gender Medicine Research ‘GENESIS’, Research center, Naples, Italy; Cardiac Unit, AORN A Cardarelli, Naples, Italy; Department of Clinical, Internal, Anesthesiology and Cardiovascular Sciences, Sapienza University of Rome, Viale del Policlinico 155, Rome 00161, Italy; Department of Cardiology, University of Milano School of Medicine, San Paolo Hospital, Milano, Italy; Department of Cardiology, University of Milano School of Medicine, San Paolo Hospital, Milano, Italy; Department of Cardiology and Jessa & Science, Jessa Hospital, Hasselt, Belgium; Faculty of Medicine and Life Sciences/LCRC, Hasselt University, Diepenbeek, Belgium; Health Sciences, UniCamillus-Saint Camillus International University, Rome, Italy; Cardiology Department, Fondazione Policlinico Tor Vergata, Roma, Italy; Department of Cardiology, San Luca Hospital, Istituto Auxologico Italiano, IRCCS, Milan, Italy; Department of Advanced Biomedical Sciences, Italian Society of Cardiology, Federico II University of Naples, Naples, Italy; Department of Advanced Biomedical Sciences, Italian Society of Cardiology, Federico II University of Naples, Naples, Italy; Cardiology Department, Fondazione IRCCS Policlinico S Matteo, Pavia, Italy; Cardiology Department, Fondazione IRCCS Policlinico S Matteo, Pavia, Italy; Cardiovascular Translational Laboratory, IRCCS San Raffaele di Roma,Rome, Italy

**Keywords:** Right ventricular dysfunction, SGLT-2i treatment, Echocardiography, Longitudinal function, Tricuspid regurgitation

## Abstract

**Background:**

Right ventricular (RV) dysfunction (RVD) represents a power independent prognostic factor in patients with chronic heart failure (CHF); poor evidence exists about the effect of current guideline directed medical therapy on RVD. We investigated whether dapagliflozin improves right ventricular function, right ventricular–pulmonary arterial coupling and pulmonary artery systolic pressure compared with standard therapy in CHF.

**Methods:**

This is a prospective observational registry with propensity score analysis investigating RV function and right heart failure (RIVED), focused on the effects of Dapagliflozin. Efficacy measures were intra and inter-groups changes from baseline to 6 months in RV end diastolic diameter (RVEDD), tricuspid annular peak systolic excursion (TAPSE), RV global longitudinal strain (RVGLS), fractional area changes (FAC), RV systolic tissue doppler wave (S’), PASP values, TAPSE/PASP, and tricuspid regurgitation (TR). Secondary endpoints were N-terminal pro B-type natriuretic peptide (NTproBNP) levels changes and combined adverse events rate of mortality and hospitalization at 180 days.

**Results:**

The final population analysed was of 142 patients, in a balanced dataset consisting of 71 treated with standard HF therapy plus dapagliflozin (Dapa group) and 71 treated with standard therapy without dapagliflozin (control group).Median age was 78 [75–84] years old and 62% of patients were men. Patients in dapa group were younger [76 (66–82) vs 82 (74–88) years old; *P* < .001] and more frequently affected by diabetes mellitus (44% vs 22%; *P* = .002) compared with control group. No significant differences were observed between the two groups in terms of gender, renal function, NTproBNP and NYHA class. Over the 6 months follow-up, the Dapa group showed a significant improvement of RV function compared with controls (TAPSE +1.03 mm, *P* < .001 and S’ +0.48 cm/s, *P* = .006); whereas no significant differences between-groups were observed for FAC (*P* = .16). RV GLS at follow-up was significantly improved in the Dapagliflozin group compared with controls (adjusted mean difference −1.54%, *P* < .001) as well as of the pulmonary-arterial coupling (TAPSE/PASP adjusted differences ranging from +0.07 to +0.17, *P* = .025). In patients with PH, a significant decrease in PASP [30 (25–34) vs 37 (31–45) mmHg, *P* < .001] associated with a RVGLS significant improvement [−20 (−22 to −19) vs −19 (−20 to −17) *P* < .001] in the Dapagliflozin group were observed. TAPSE/PASP ratio was significantly improved in Dapagliflozin group with respect to the control group [0.67 (052–0.78) vs 0.51 (0.42–0.61), *P* < .001]. Secondary endpoints analysis revealed that NT-proBNP levels were significantly improved from baseline to follow-up only in dapaglifozin group (from 938 to 726 pg/mL *P* = .013). The rate of 180-days adverse events was significantly higher in control group with respect to Dapaglifozin group (21% vs 8%, *P* < .01).

**Conclusions:**

In a real-world HF cohort, dapagliflozin was associated with improved RV morphology and function, lower PASP, and better RV–PA coupling, suggesting a direct haemodynamic and RV-targeted benefit beyond LV remodelling.

**Trial Registration:**

(ClinicalTrials.gov Identifier: NCT06002321)

## Graphical Abstract

**Graphical Abstract:**
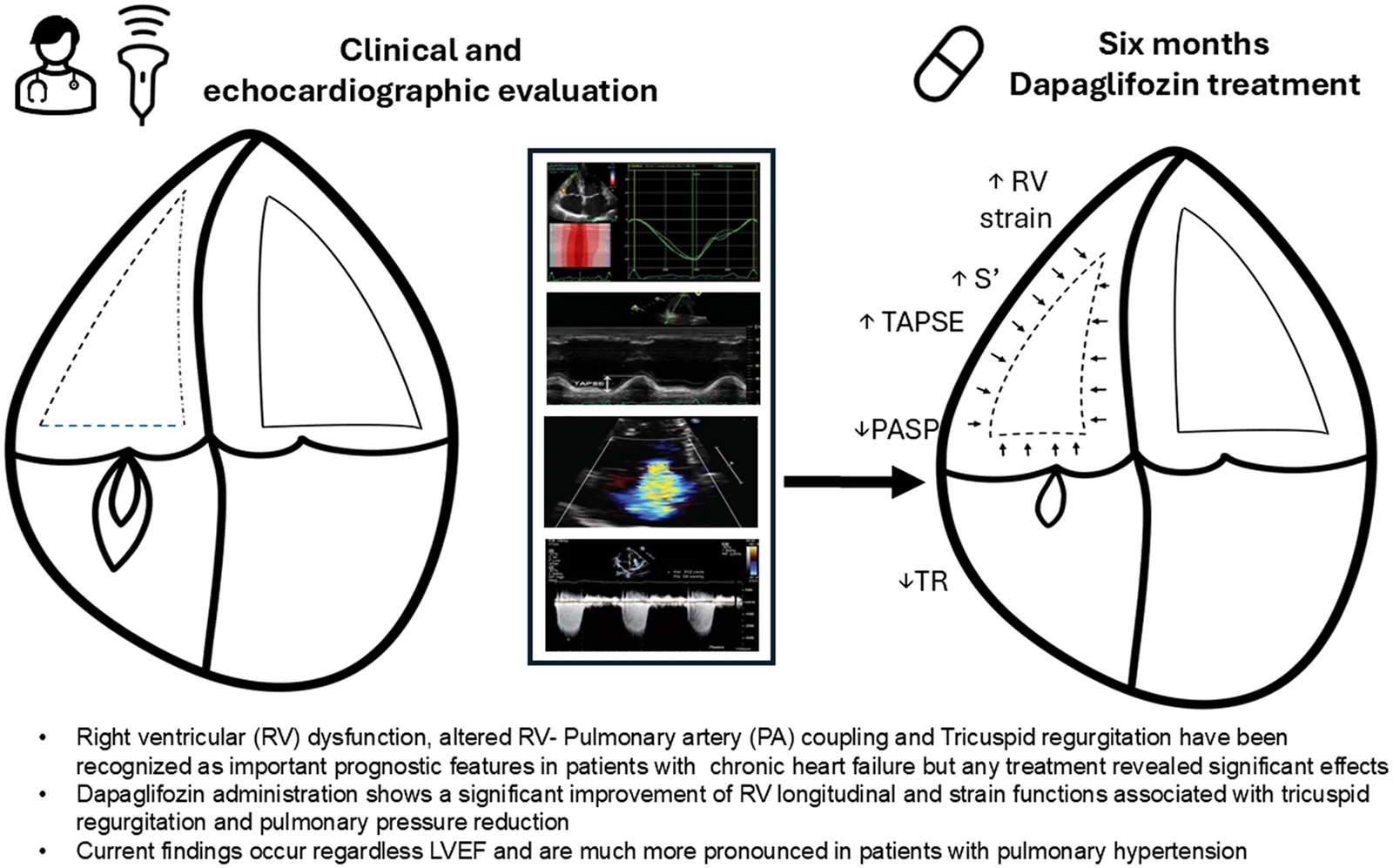

## Introduction

Right ventricular dysfunction (RVD) and pulmonary hypertension (PH) are two important features in patients affected by left side heart failure (HF), associated with an increased risk of adverse event and poor quality of life.^1,2^ Although RVD has been universally recognized as one of the most important prognostic factors in chronic HF, current HF guidelines not providing a specific strategy.^3^ However, prevalence and type of RVD, as well as the impact on the disease, could vary among different left ventricular ejection fraction (LVEF) thresholds and HF subtypes.^4^ For instance, in subjects affected by HF with reduced ejection fraction (HFrEF) and those with more advanced HF stages, the RVD is a concomitant feature; on the other hand, in patients with a less severe stage, RVD degree could vary according to post-capillary pulmonary pressure, vascular resistances, and myocardial dysfunction extension that are responsible for primitive cardiac disorder.^5–8^

Behind the different echocardiographic parameters employed, current HF treatment showed relevant impact on LV remodelling and function, but scarce evidence has been reported on RV function and tricuspid regurgitation.^9^

In this context, Sodium-Glucose Cotransporter-2 inhibitors (SGLT-2i) represent a new class of agents recently endorsed for the treatment of HF across the whole spectrum of LVEF and are now recommended as the first-class option in all HF patients regardless NYHA class, HF aetiology, and history.^10–12^ Recent evidence demonstrated a haemodynamic impact on mean pulmonary pressure and wedge pressure in patients investigated with invasive monitorization. In particular, it is unclear whether SGLT2i improve RV contractile indices and RV–PA coupling in routine HF care, outside invasive haemodynamic studies.^13^

Thus, we aimed to assess the impact of dapagliflozin on RV function, PASP, RV–PA coupling, and TR over 6 months in a stable, optimally treated chronic HF population.

## Methods

The present investigation is an exploratory propensity score analysis focused on the effects of Dapagliflozin (Dapa group) at 6 months follow-up obtained from a national multicentre observational trial investigating RV function and right heart failure. The RIght VEntricular Dysfunction in chronic Heart Failure (RIVED-HF) study is a multi-centre observational prospective registry endorsed from Italian Society of Cardiology aimed to investigate the association between right ventricular dysfunction and sign and symptoms of Right HF (RHF) (ClinicalTrials.gov Identifier: NCT06002321).^14^

The full study design has been elsewhere described. In brief, ambulatory patients with stable HF and optimized treatment, regardless LVEF and NYHA class, are involved in the present registry, with a visit and/or virtual visits every 6 months for a mean period of 3 years.

### Study protocol

The demographic, NYHA classification, biochemical and laboratory data, including NTproBNP, index of renal and liver function, electrolytes, glomerular filtration rate (GFR-calculated using the Modification of Diet in Renal Disease formula) have been collected together with information about HF aetiology, medical history, associated diseases, risk factors, and drug administration during initial visit and repeated every 6 months. Clinical and congestion evaluation including dyspnoea, orthopnoea, jugular vein reflex, cave vein dilatation, lung rales, additional heart murmur, peripheral oedema have been assessed according to Gheorghiade assessment.^15^

For the present analysis, the primary aims were the changes from baseline to 6 months follow-up in echocardiographic measurement of RV morphology and function, and a comparison between patients with SGLT2i and patients without. Patients who were not treated with dapaglifozin represented the control group of our study. Thus, we included patients evaluated before SGLT-2i approval for HF or with dapaglifozin intolerance or controincation. [Supplementary Figure S2]—As secondary end points, hospitalization and mortality for cardiovascular causes were considered.

### Exclusion Criteria

Patients with non-cardiogenic dyspnoea related to systemic lung diseases, pulmonary embolism, primitive pulmonary hypertension (PH), have been excluded, as well as primitive significant valvular diseases, and infiltrative hypertrophic muscular diseases. In addition, patients with acute HF, in non-optimized therapy, or with a diagnosis performed in less than three months have been excluded. Finally, patients with neoplastic, systemic, and inflammatory diseases, renal or hepatic diseases, or waiting for transplantation, have been excluded.

### Enrolment procedure

All patients affected by chronic HF have been consecutively enrolled in a prospective modality. HF diagnosis was based on ESC Guidelines recommendations, and patients have been categorized according to LVEF in HFpEF (LVEF >50%), HFmEF (LVEF ranges from 40 to 49%), and HFrEF (LVEF < 40%).^10^ For the purpose of the present analysis, according with more recent opinions and future classification, we consider HFmEF and HFrEF together.

### Physical examination

Patients were evaluated by two distinct physicians. In all patients, NYHA class, clinical characteristics, heart failure aetiology and phenotype, risk factors, comorbidities, congestion, and perfusion status, have been evaluated. In addition, patients have been classified four stages according with RHF classification.^16^ In the present analysis all patients were in stage C of RHF.

### Laboratory test

The following laboratory parameters have been measured at the initial screening and at 6 months: blood count, C-reactive protein, NTproBNP or BNP, troponin, creatinine, sodium, potassium, urea, glycaemia, transaminases, osmolarity.

### Echocardiography

A comprehensive 2-D echocardiography exam with doppler and tissue doppler imaging (TDI) analysis was performed. All the examinations were performed by physicians according to the instructions provided by the American Society of Echocardiography.^17^ The main measurements were independently reviewed by two distinct physicians; systolic and diastolic left ventricular volumes and ejection fraction was determined using apical two- and four chamber views by Simpson biplane formula. Left ventricular diastolic function was assessed by Pulsed Doppler *trans* mitral flow velocity will be used to assess early diastolic velocity (E wave), late diastolic velocity (A wave) their ratio (E/A), deceleration time (DT) of E wave. Early diastolic myocardial velocity (e′) was obtained by TDI from the apical 4 chamber view using 1.5 mm sample volume at septal level and the E/e′ ratio was measured to classify diastolic function.^18^

### RV function assessment

Tricuspid annular peak systolic excursion (TAPSE) was calculated by placing the M-mode cursor laterally to the tricuspid annulus.^19^ Pulmonary artery systolic pressure (PASP) was estimated by continuous Doppler at the tricuspid valve level, as the sum of 4× (peak velocity of TR velocity) 2 and the estimate of right atrial pressure (RAP) based on inferior vena cava diameter and collapsibility. S wave (S’) was obtained by TDI analysis. Patients were categorized as having high PASP if the PASP estimate was >30 mmHg. The cut-off for RV dysfunction were TAPSE of ≤16 mm and S’ < 9 cm/sec.^20^ Tricuspid regurgitation (TR) degree has been assessed grading from 1 to 4 according to regurgitation severity (mild/moderate/severe/massive-torrential), through vena contracta and PISA criteria.^21^ The right ventricular end-diastolic diameter (RVEDD) was measured at basal level below tricuspid anulus and at mid RV level at moderator band insertion with free RV wall in apical four chamber view during late diastolic phase. Fractional area change (FAC) has been calculated by end diastolic and end systolic RV area calculation based on European association cardiovascular imaging (EACVI) criteria.^17^ Finally RV free wall and interventricular septum strain measurements were calculated.^22^

Primary endpoints were changes from baseline to 6 months in RV longitudinal function (TAPSE, S′, RVGLS) and RV–PA coupling (TAPSE/PASP).

Secondary endpoints included changes in PASP, TR grade, NT-proBNP, and clinical outcomes (HF hospitalization and CV mortality).

### Statistical analysis

Normality of continuous variables was assessed using the Shapiro–Wilk test. None of the continuous variables showed a normal distribution; therefore, results are presented as median and interquartile range (25th and 75th percentiles).

To improve baseline comparability between treated and untreated patients, a propensity score modelling, trimming, and sample balancing procedure was performed. This procedure resulted in a balanced dataset consisting of 71 treated and 71 untreated patients.

To reduce baseline differences between treatment groups, we estimated a propensity score (logistic regression) including RV and pulmonary variables (PASP, pulmonary hypertension, TAPSE, S′, FAC, RVEDD, RVGLS, TAPSE/PASP, and TR grade). Patients were restricted to the region of common support. A balancing procedure yielded 71 dapagliflozin-treated and 71 untreated patients with improved covariate balance (standardized mean differences substantially reduced for all variables). Before adjustment, several baseline variables showed moderate to substantial imbalance between groups (e.g. PASP SMD = 0.629 and pulmonary hypertension SMD = 0.565). After restriction to the common support region and balancing, the final analytic cohort included 71 treated and 71 untreated patients, with a marked reduction in baseline differences across all propensity variables (e.g. PASP SMD reduced to 0.329 and pulmonary hypertension SMD to 0.225), indicating improved comparability between treatment groups.

Between-group comparisons (treated and untreated patients) were performed using the Wilcoxon rank-sum test, while within-group changes from baseline to 6-month follow-up were evaluated using the Wilcoxon signed-rank test for paired data. Changes in categorical variables from baseline to 6-month follow-up, including NYHA functional class and TR class were analyzed using the Stuart–Maxwell test for paired categorical data. All categorical analyses were performed separately for treated and untreated patients.

Prespecified subgroup analyses were conducted according to left ventricular ejection fraction (HFrEF vs HFpEF) and the presence of pulmonary hypertension at baseline, applying the same non-parametric statistical approach.

To further account for potential residual imbalance after the propensity score procedure, between-group comparisons at 6-month follow-up were also performed using analysis of covariance (ANCOVA) models adjusted for the corresponding baseline value of each variable.

This approach was also applied to NT-proBNP levels, which were analysed both on the original scale and after logarithmic transformation as a sensitivity analysis, given their skewed distribution.

Robust standard errors were used in regression models to account for potential deviations from model assumptions. Analyses were performed on complete-case data; no imputation for missing values was applied.

## Results

### Baseline clinical characteristics

From the RIVED population, 213 patients were initially selected for propensity score modelling; however, 28 did not meet echocardiographic and clinical criteria or were excluded for poor acoustic window, 21 were lost during follow-up, and 22 were excluded after propensity score adjustment (*Figure 1*). As a result, the final sample was of 142 patients, in a balanced dataset consisting of 71 treated with standard HF therapy plus dapagliflozin (Dapa group) and 71 treated with standard therapy without dapagliflozin (control group). The baseline characteristics of the total population and of the final sample included are showed in *Table 1*.

**Figure 1:**
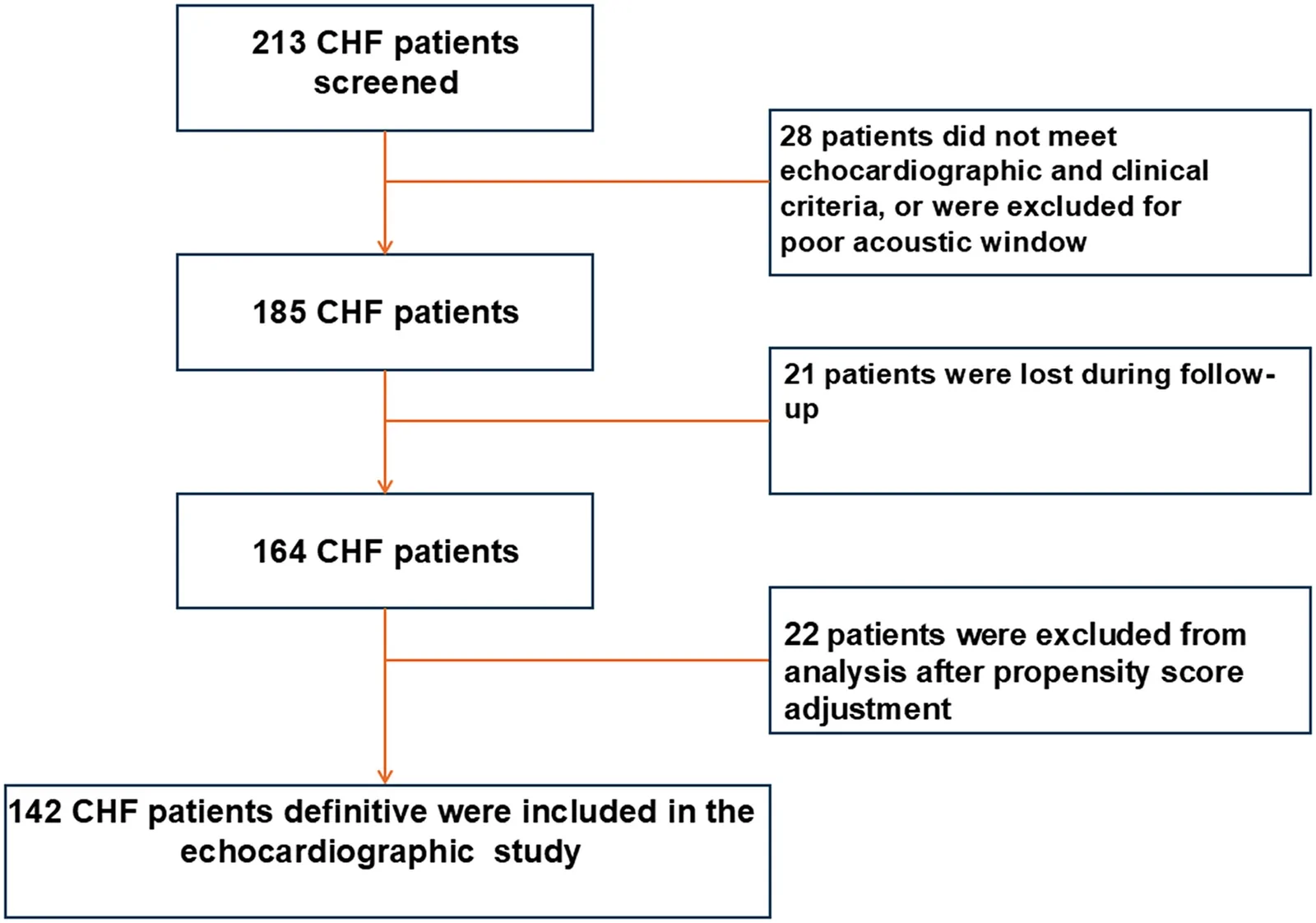
Timing frame and consort diagram of initial included population and final sample size

**Table 1:** Differences in baseline clinical and laboratory characteristics among patients who were treated with dapaglifozin vs patients who were not at first HF visit

	All patients (*n* = 142)	Dapagifozin (*n* = 71)	No dapaglifozin (*n* = 71)	*P*-value
**Age (years)**	78 (75–84)	76 (66–82)	82 (74–88)	<.001
**Gender Male (%)**	62	68	52	<.34
**Laboratory data before the treatment**				
Creatinine (mg/dL)	1.19 (.96–1.52)	1.19 (1.04–1.52)	1.16 (0.90–1.53)	.367
eGFR (mL/min/1.73 m^2^)	58.5 (44–68)	60 (45–67)	56 (40–70)	.36
NTproBNP (pg/mL)	1031 (546.5–2000.5)	1082.5 (559.7–1992)	1002 (537–2031)	.825
NYHA class 2/3 (%)	53/19	42/22	65/14	.07
**Risk factors (%)**				
Diabetes Mellitus	35	44	22	.002
Hypertension	56	52	64	.104
Dyslipidaemia	54	56	52	.667
**Medical history**				
**Atrial fibrillation (%)**	36	32	41	.255
**Atrial flutter (%)**	2	2	3	.007
**Ventricular arrhythmias (%)**	3	5	2	.333
**Presence of PM (%)**	14	10	17	.208
**Presence of ICD (%)**	16	20	12	.07
**Presence of CRT-D (%)**	10	12	8	.064
**Previous MI (%)**	37	40	35	.107
**CKD (%)**	30	31	29	.893
**ESRD (%)**	13	10	17	.208
**COPD (%)**	14	12	19	.27
**HF Home therapy**				
**ACEis (%)**	38	37	39	.86
**ARBs (%)**	26	20	33	<.05
**ARNI (%)**	33	39	27	<.05
**MRAs (%)**	51	53	50	.75
**Diuretics (%)**	76	74	80	.435
**Β-blockers (%)**	74	72	76	.732

The echocardiographic baseline analysis revealed similar value between dapagliflozin group compared with standard care group for LVEF [40 (34–54) vs 45 (38–53)], end diastolic left ventricle diameter (EDDLV) [56 (50–61) vs 55 (49–62) mm], end systolic left ventricle diameter (ESDLV) [41 (33–49) vs 38 (34–45) mm], end diastolic left ventricular volume (EDVLV) [58 (52–70) vs 58 (52–67) ml/m2], end systolic left ventricular volume (ESVLV) [33 (25–46) vs 30 (25–42) ml/m2], E/e′ [12 (10–15) vs 11 (9–14)], and LAVI [55 (34–70) vs 50 (40–61) ml/m2]. Among RV echocardiographic data, we did not find significant difference at baseline in terms of basal end diastolic right ventricular diameter (RVEDD), PASP, TAPSE, TAPSE/PASP ratio, FAC and RV global longitudinal strain (RVGLS) (*Table 2*).

**Table 2:** Echocardiographic characteristics in patients treated with dapaglifozin vs patients who were not treated with dapaglifozin

Variables	All patients (n.142)	Dapaglifozin (n. 71)	No dapaglifozin (n.71)	*P*-value
***Echocardiography: LV***				
EDVLV (ml/m2)	58 (52–69)	58 (52–70)	58 (52–67)	.982
ESVLV (ml/m2)	31 (25–44)	33 (25–46)	30 (25–42)	.707
EDDLV (mm)	55 (49–62)	56 (50–61)	55 (49–62)	.565
ESDLV (mm)	39 (33–42)	41 (33–49)	38 (34–45)	.371
LVEF (%)	45 (35–53)	40 (34–54)	45 (38–53)	.389
LAVI (ml/m2)	51 (38–67)	55 (34–70)	50 (40–61)	.720
E/e′	12 (10–14)	12 (10–15)	11 (9–14)	.336
***Echocardiography: RV***				
RVEDD (mm)	38 (35–42)	38 (35–41)	38 (34–43)	.622
TAPSE (mm)	19 (17–21)	18 (17–20)	19 (18–21)	.199
PASP (mmhg)	35 (30–42)	35 (30–42)	38 (33–45)	.074
TAPSE/PASP	0.51 (0.40–0.66)	0.52 (0.40–0.67)	0.51 (0.39–0.63)	.450
S’(cm/sec)	11 (10–12)	11 (10–12)	12 (10–13)	.024
FAC %	45 (39–50)	44 (39–50)	46 (40–50)	.265
RV GLS %	−19 (−17 to 21)	−19 (−18 to −21)	−19 (−16 to −21)	.356

EDDLV, end diastolic left ventricle diameter; EDVLV, end diastolic left ventricle volume; ESDLV, end systolic left ventricle diameter; ESVLV, end systolic left ventricle volume; FAC, fractional area; GLS, global longitudinal strain; LAVI, left atrial volume index; LVEF, left ventricle ejection fraction; PASP, pulmonary artery systolic arterial pressure; RV, right ventricle; RVEDD, end diastolic right ventricle diameter; S’, systolic wave velocity; TAPSE, tricuspid annular peak systolic excursion.

### RV analysis according with treatment

The longitudinal analysis of RV echocardiographic variables from baseline to 6 months confirmed a more pronounced improvement in Dapagliflozin group compared with control group. We observed a significant RVEDD reduction [38 (35–41) vs 37 (34–40) mm, median difference 0; *P* < .005], a significant increase in TAPSE [18 (17–20) vs 19 (18–21) mm, median difference 0; *P* < 0001], S’ [11 (10–12) vs 11 (10–12) cm/sec, median difference 0; *P* < .012], and FAC [44 (39–50) vs 45 (40–49)%, median difference 0.10; *P* < .004], as well as a RV GLS improvement [−19 (−21–17) vs 20 (−22–18), median difference −1; *P* < .001]. Finally, also the TAPSE/PASP ratio increased [0.52 (040–0.67) vs 0.64 (0.48–0.76), median difference 0.07; *P* < .001] (*Table 3*). In the control group, at 6 months we observed a slight increase of RVEDD [38 (34–43) vs 38 (35–43) mm, median difference 0; *P* = .03], a significant decrease in TAPSE [19 (18–21) vs 18 (17–21) mm, median difference 0; *P* < .003] and a stable value of PASP [38 (33–45) vs 38 (32–45) mmHg, median difference 0; *P* = .97]. These findings were associated with a significant reduction in RVGLS [−29 (−21–16) vs −19 (−18–16)], and S’ [12 (10–13) vs 11 (10–12) cm/sec, median difference 0; *P* < .02], with no differences in FAC [46 (40–50) vs 45 (40–50)%, median difference 0; 0.37], and TAPSE/PASP ratio [0.51 (0.39–0.63) vs 0.49 (0.38–0.60), median difference −0.03; *P* 0.171] (*Table 4* and *Figure 2*).

**Figure 2:**
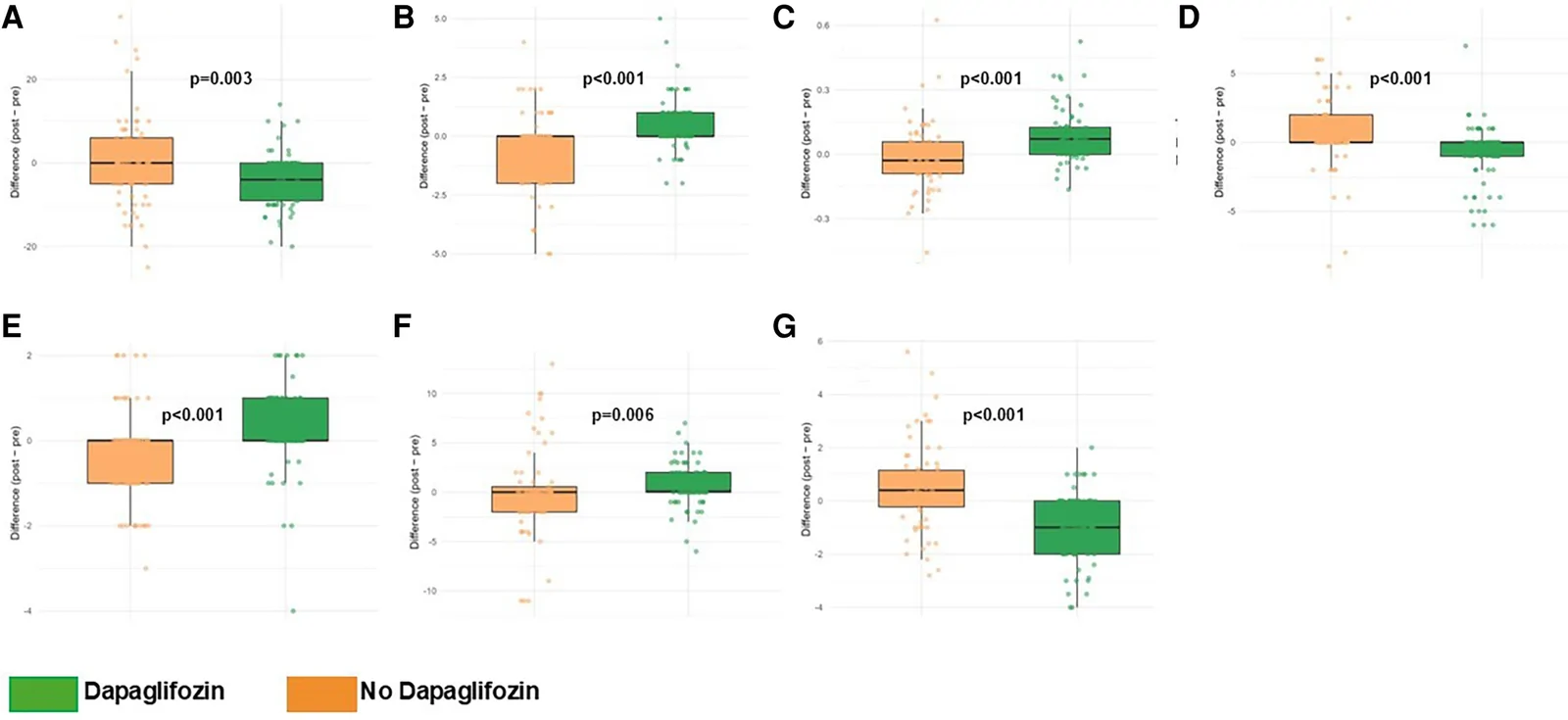
RV echocardiographic assessment of patients in dapaglifozin group vs control group from baseline to 6 months follow-up period revealing an improvement in RV function in active arm

**Table 3:** Median paired difference among echocardiographic variables of RV at baseline and at 6 months in group treated with dapaglifozin

Variables	Baseline	6 months	Median differences	*P*-value
***Echocardiography: RV***				
RVEDD (mm)	38 (35–41)	37 (34–40)	0 (−1 to 0)	.005
TAPSE (mm)	18 (17–20)	19 (18–21)	0 (0–1)	<.001
PASP (mmhg)	35 (30–42)	30 (25–36)	−4 (−9–0)	<.001
TAPSE/PASP	0.52 (0.40–067)	0.64 (0.48–0.76)	0.07 (0.00–0.12)	<.001
S’ (cm/sec)	11 (10–12)	11 (10–12)	0 (0–1)	.012
FAC%	44 (39–50)	45 (40–49)	0.10 (0.00–2.00)	.004
RV GLS%	−19 (−21 to −17)	−20 (−22 to −18)	−1 (−2–0)	<.001

FAC, fractional area; PASP, pulmonary artery systolic pressure; RV, right ventricle; RVEDD, end diastolic diameter of right ventricle; RVGLS, right ventricle global longitudinal strain; S’, systolic wave; TAPSE, tricuspid annular peak systolic excursion.

**Table 4:** Median paired difference among echocardiographic variables of RV at baseline and at 6 months in group not treated with dapaglifozin

Variables	Baseline	6 months	Median differences	*P*-value
***Echocardiography: RV***				
RVEDD (mm)	38 (34–43)	38 (35–43)	0 (0–2)	.026
TAPSE (mm)	19 (18–21)	18 (17–21)	0 (−2–0)	.003
PASP (mmhg)	38 (33–45)	38 (32–45)	0 (−5–6)	.976
TAPSE/PASP	0.51 (0.39–0.63)	0.49 (0.38–0.60)	−0.03 (−0.09–0.06)	.171
S’ cm/sec	12 (10–13)	11 (10–12)	0 (−1 to 0)	.019
FAC%	46 (40–50)	45 (40–50)	0 (−2 to 0.54)	.369
RV GLS%	−19 (−21 to −16)	−18 (−20 to −16)	0.4 (−0.23 to 1.15)	.012

FAC, fractional area; PASP, pulmonary artery systolic pressure; RV, right ventricle; RVEDD, end diastolic diameter of right ventricle; RVGLS, right ventricle global longitudinal strain; S’, systolic wave; TAPSE, tricuspid annular peak systolic excursion.

At 6 months, ANCOVA adjusted for baseline values showed significantly higher TAPSE and S’ in the Dapagliflozin group compared with controls (adjusted mean difference +1.03 mm, *P* < .001 and +0.48 cm/s, *P* = .006, respectively), whereas no significant differences between-groups were observed for FAC (*P* = .16). RV GLS at follow-up was significantly improved in the Dapagliflozin group compared with controls (adjusted mean difference −1.54%, *P* < .001). A significant treatment-by-baseline interaction was observed for the TAPSE/PASP ratio (*P* for interaction = .001), indicating that the treatment effect varied according to baseline values. At 6 months, TAPSE/PASP ratio was significantly higher in the Dapagliflozin group, with adjusted differences ranging from +0.07 to +0.17 across increasing baseline levels (all *P* = .025). PASP at follow-up was significantly lower in the Dapagliflozin group compared with controls, with a significant interaction with baseline PASP (*P* for interaction = .047), with a greater treatment effect in patients with higher baseline values. Finally, RVEDD at 6 months was significantly lower in the Dapagliflozin group compared with controls (adjusted mean difference −1.54 mm, 95% CI −2.31 to −0.76, *P* < .001), after adjustment for baseline values (*Table 5*).

**Table 5:** Adjusted between-group differences at 6-month follow-up (ANCOVA)

Variable (6 months)	Adjusted difference (Dapa vs control)	95% CI	*P* value
EDDRV (mm)	−1.54	−2.31 to −0.76	<.001
TAPSE (mm)	+1.03	+0.58 to +1.48	<.001
PAPS (mmHg)	—	—	<.001^a^
TAPSE/PAPS	—	—	<.001^a^
S′	+0.48	+0.14 to +0.82	.006
FAC	+0.91	−0.34 to +2.17	.156
RV GLS	−1.54	−2.00 to −1.07	<.001

Between-group comparisons at 6 months were performed using analysis of covariance, adjusted for baseline values of the corresponding variables. Robust standard errors (HC3) were applied. Positive values indicate higher measurements in the Dapa group compared with controls, whereas negative values indicate lower values.

^a^A significant treatment-by-baseline interaction was observed; therefore, a single adjusted estimate is not reported. *P* value refers to the overall treatment effect accounting for the interaction term.
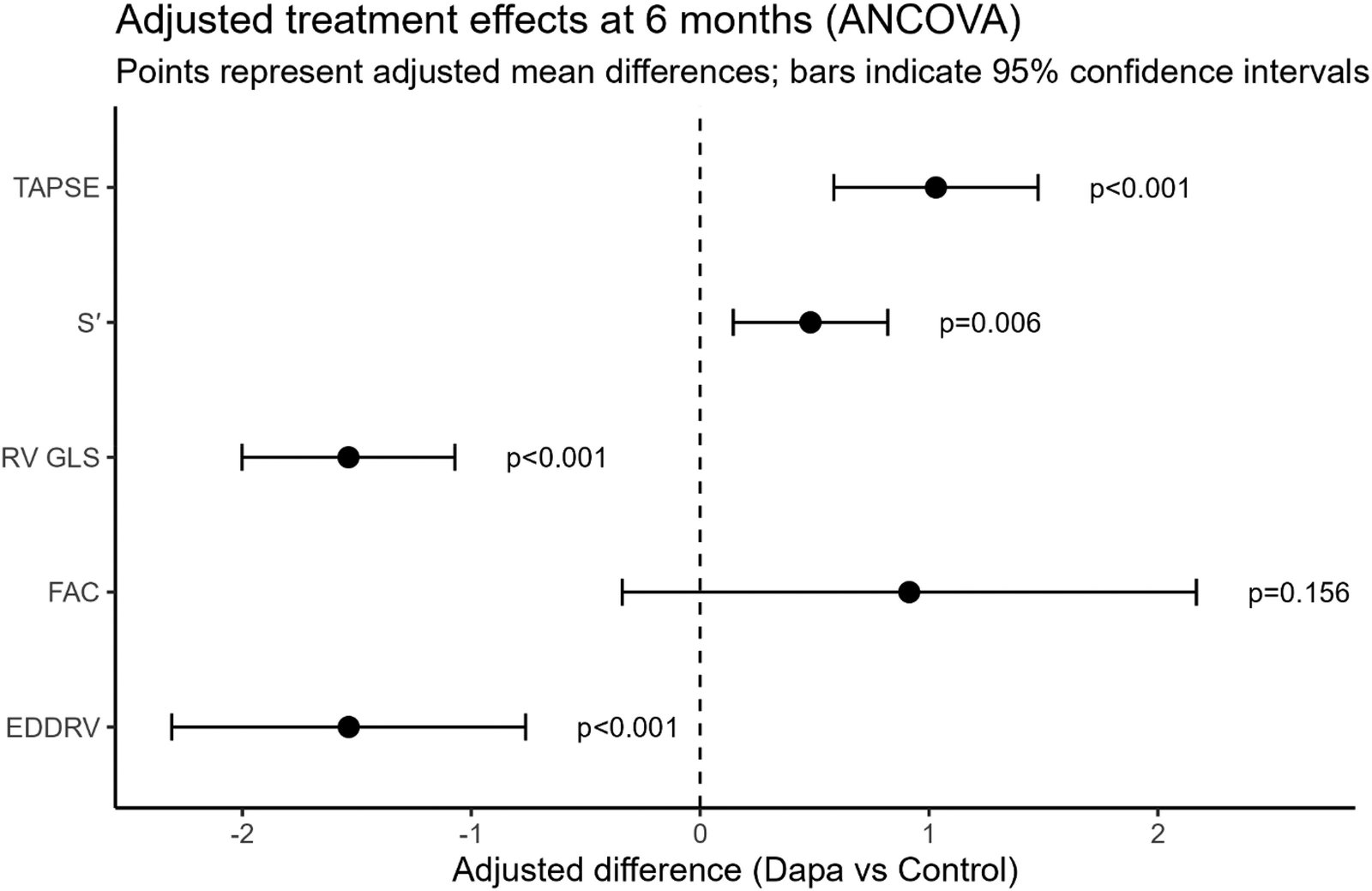

In Dapagliflozin patients, the distribution of TR grade showed a change from baseline to follow-up with borderline statistical significance (*P* = .05). Specifically, 22.8% of patients showed an improvement in TR grade, 71.9% remained stable, and 5.3% experienced a worsening at follow-up. In the control group, no statistically significant changes in TR grade distribution were observed (*P* = .376): 22.1% of patients showed an improvement in TR grade, 63.2% remained stable, and 14.7% experienced a worsening at follow-up (*Figure 3*).

**Figure 3:**
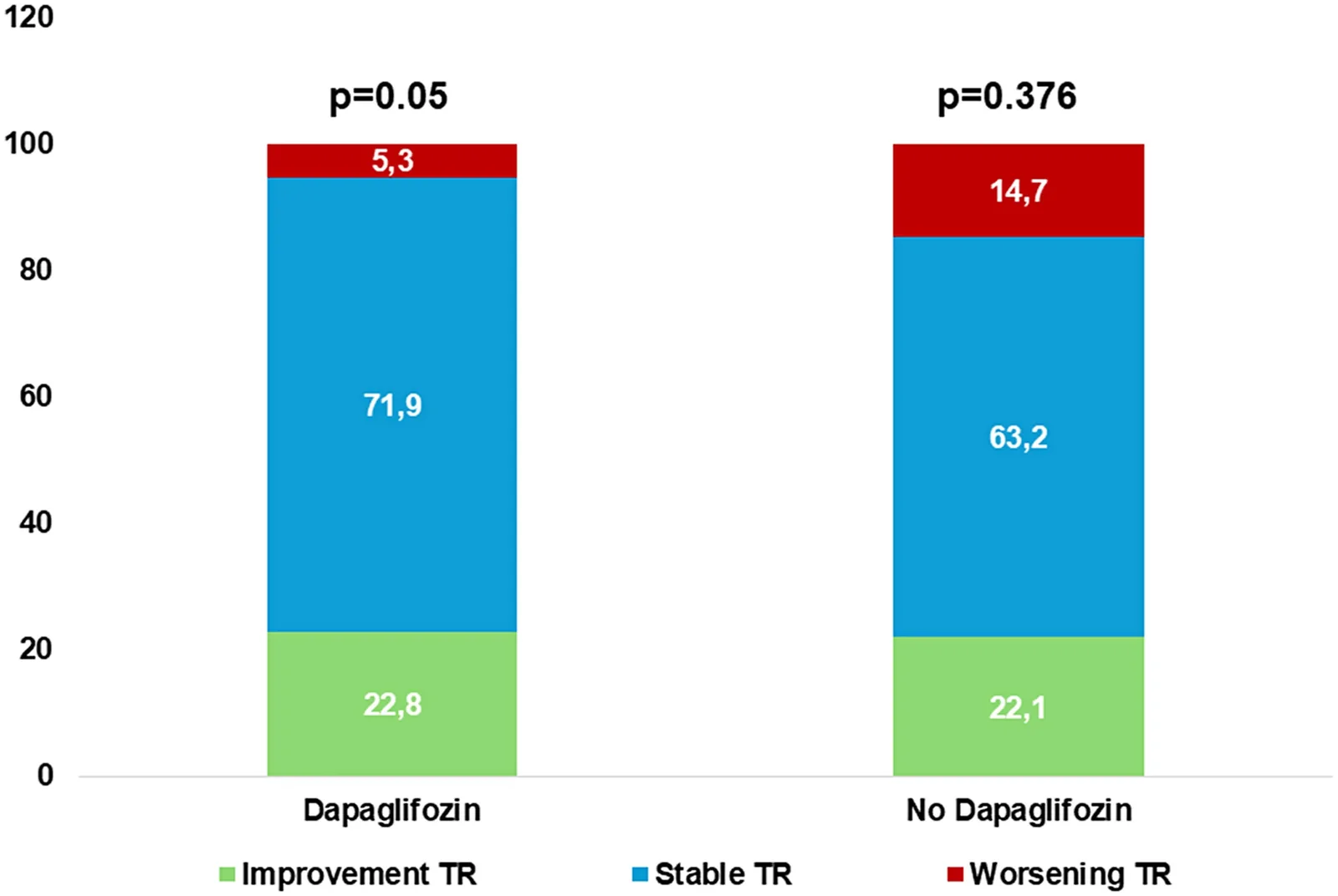
Changes in tricuspid regurgitation degree from baseline to 6 months in two groups revealing a substantial improvement of TR in dapa group

### RV analysis in HFrEF vs HFpEF

Dividing our population according to LVEF cut-off more or <50%, we found 92 patients with HFrEF and 50 affected by HFpEF. In HFrEF patients, we observed a significant lower RVEDD [37 (35–41) vs 40 (36–44) mm; *P* < .04] at 6 months in Dapagliflozin group; in addition, a decrease in PASP [30 (24–50) vs 40 (30–70) mmHg; *P* < .001] and a significant improvement of TAPSE [19 (18–21) vs 18 (16–20); mm *P* < .02], RVGLS [−20 (−18 to −21) vs −18 (−16 to −20) *P* < .002] and TAPSE/PASP [0.63 (0.44–0.78) vs 0.47 (0.38–0.57); *P* < .001] were observed in the dapagliflozin group at 6 months. No significant differences were found in FAC [42 (40–48) vs 44 (35–50) % *P* .78] and S’ [11 (10–12) vs 11 (10–12) cm/sec; *P* .76] between the two groups.

In HFpEF patients, we found a significant reduction of PASP [30 (27–35) vs 35 (34–45) mmHg, *P* = .003], a significant improvement in RVGLS [−21 (−22 to −18) vs −19 (−20 to −17) *P* = .008], and a borderline improvement of the TAPSE/PASP [0.64 (0.50–0.76) vs 0.54 (0.44–0.63) *P* < .055]. No further differences for TAPSE, FA, RVEDD, and S’ between Dapagliflozin and control groups were observed (*Table 6*).

**Table 6:** Right ventricle (RV) analysis according to left ventricle ejection fraction (LVEF) in dapa vs control groups

Variables	LVEF<50%			LVEF>50%		
Echocardiography	Dapa group (46)	Control group (46)		Dapa group (25)	Control group (25)	
EDDRV mm	37 [35–51]	40 [36–44]	*P* = .03	36 [34–40]	37 [35–40]	*P* = .47
TAPSE mm	19 [18–21]	18[16–20]	*P* = .02	19 [17–21]	20 [18–22]	*P* = .24
PAPS mmHg	30[25–50]	40 [35–65]	*P* < .001	30 [27–35]	35 [34–45]	*P* < .003
TAPSE/PAPS	0.63 [0.44–0.68]	0.47 [0.38–0.57]	*P* < .001	0.64 [0.50–0.76]	0.54 [0.44–0.63]	*P* < .05
S’cm/sec	11 [10–12]	11 [10–12]	.76	11[10–12]	12 [10–13]	*P* = .72
FA %	42[40–48]	44[35–50]	.78	46 [42–51]	46 [44–53]	*P* = .29
RV GLS%	−20 [−18 to −21]	−18 [−16 to −20]	*P* < .003	−21 [−18 to −22]	−19 [−17 to −20]	*P* < .008

EDDRV, end diastolic diameter of right ventricle; FA, fractional area; PAPS, pulmonary artery systolic pressure; RV, right ventricle; RVGLS, right ventricle global longitudinal strain; S’, systolic wave; TAPSE, tricuspid annular peak systolic excursion.

### RV analysis in PH vs non-PH

According to the presence of baseline PH, defined as PASP >30 mmHg, 123 patients were PH whereas only 19 subjects did not.

In patients with PH, a significant decrease of PASP [30 (25–34) vs 37 (31–45) mmHg, *P* < .001] associated with a RVGLS significant improvement [−20 (−22 to −19) vs −19 (−20–17) *P* < .001] at 6 months in the Dapagliflozin group were observed. Additionally, TAPSE/PASP ratio was significantly improved in Dapagliflozin group [0.67 (052–0.78) vs 0.51 (0.42–0.61) *P* < .001]. No differences for TAPSE, FAC, S’, and RVEDD was showed. (*Figure 4*) In patients without PH, a significant improvement in TAPSE/PASP [0.41 (0.38–0.63) vs 0.35 (0.26–0.38) *P* = .04] and RV GLS [−18 (−20 to −17) vs −16 (−17 to −15) *P* = .06] were observed in the Dapa arm. No other significant differences in RV echocardiographic variables within the two groups without PH were found.

**Figure 4:**
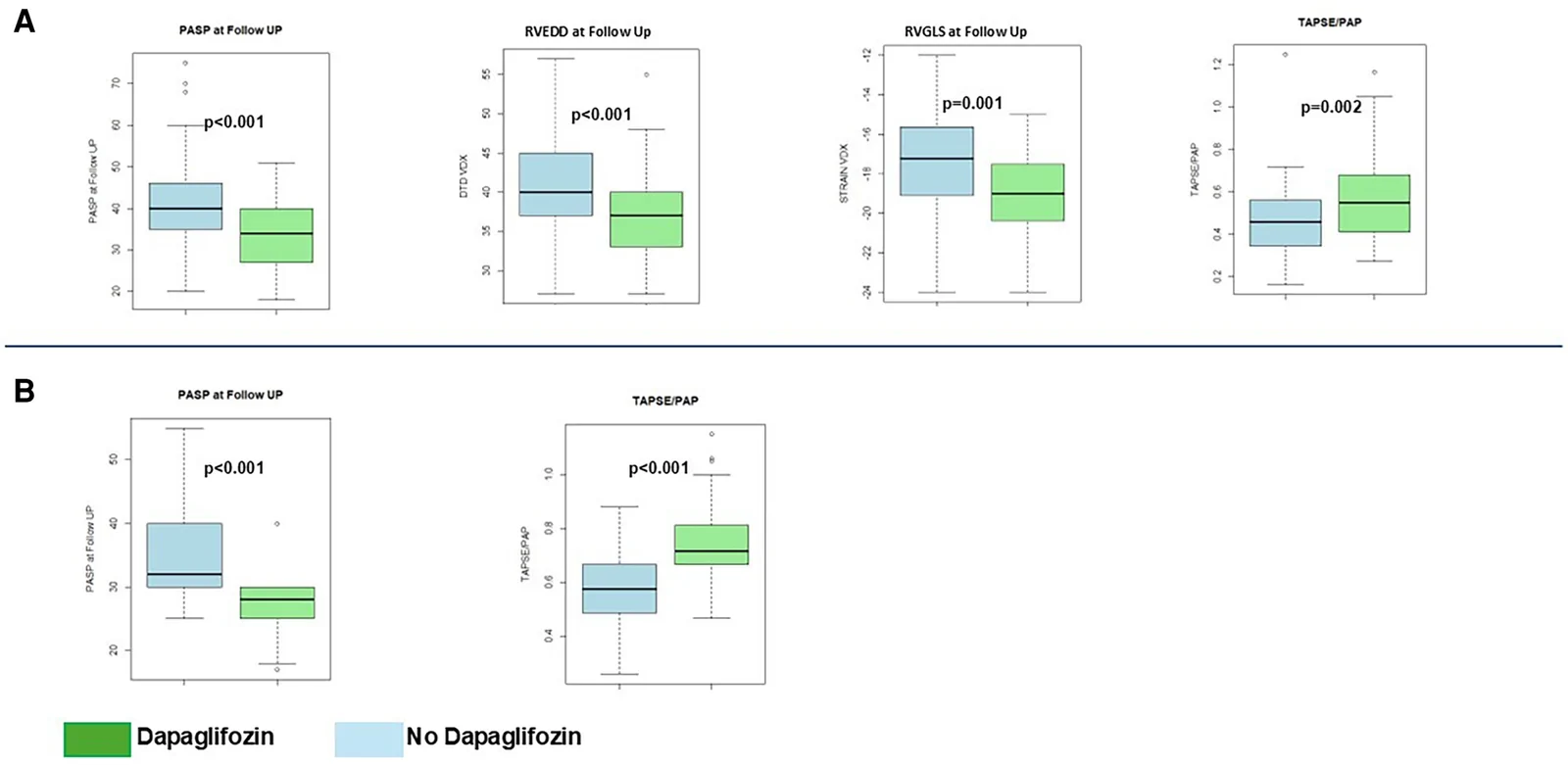
Echocardiographic analysis of RV in patients with increased pulmonary systolic pressure (PASP) defined as >30 mmHg in dapa group (A) vs control group (B)

### Co-primary outcome

Overall, 14.3% of Dapagliflozin patients showed an improvement in NYHA class, 64.3% remained stable, and 21.4% experienced a worsening at follow-up. (*P* = .01) In control group, 11.3% of control patients showed an improvement in NYHA class, 64.8% remained stable, and 23.9% experienced a worsening at follow-up, resulting in no statistically significant changes in NYHA class distribution between baseline and follow-up (*P* = .122) (Supplementary Figure S1).

NT-proBNP levels were statistically improved between baseline and follow-up (from 938 to 726 pg/mL *P* = .013, with a median absolute decrease of 87.5 units), corresponding to a median relative reduction of 17.7% in Dapagliflozin Group, whereas control group did not demonstrate significant differences (from 1540 to 1401 pg/mL). Finally, combined adverse event rate of mortality and hospitalization was 21% in control group vs 8% in Dapagliflozin group (*P* < .01).

## Discussion

The RV dysfunction is a recognized prognostic feature in chronic HF and its assessment has been emphasized in the most recent American and European Heart Failure Guidelines.^3,23^ A recent scientific statement of heart failure association (HFA) highlighted the beneficial effect of SGLT2Is treatment in acute and chronic heart failure irrespective of LVEF.^24^ The diuretic effect together with the metabolic changes in cardiomyocytes, lead to the reduction of pulmonary pressures and the LV remodelling.^24^ Indeed, a recent analysis, including 104 patients with moderate to severe functional mitral regurgitation (FMR), showed that addition of Dapaglifozin reduced FMR degree.^25^ This effect could be related to the LV filling pressure improvement and the reverse remodelling which contribute to the reduction of HF hospitalization and mortality.^25^ To date, no convincing data exists about the effects of current guidelines directed medical therapy (GDMT) on RVD and remodelling. To our knowledge, this is the first propensity-adjusted real-world registry analysis showing that dapagliflozin is associated with improved RV longitudinal function, reduced PASP, and better RV–PA coupling assessed by comprehensive echocardiography.

Our findings demonstrated that the addition of SGLT-2i treatment improved RV function and RV-PA coupling in patients with chronic HF. More specifically, dapagliflozin treatment showed a significant increase of RV strain, TAPSE, and S’ values resulting in better RV systolic function associated with a trend for TR and PASP reduction. Although the median change for some right ventricular parameters was equal to zero, statistically significant differences were observed. This reflects the non-parametric nature of the Wilcoxon signed-rank test, which evaluates the distribution of paired differences rather than the median value alone. Therefore, a significant *P*-value indicates that the distribution of individual changes differs from zero even when the median change itself remains unchanged. Additionally, current findings occur regardless LVEF, and are much more significative in patients with PH. (Graphical Abstract) as expected, RV functional improvement is associated with significant reduction in NTproBNP levels, NYHA class, and adverse event rate. Notably, the improvement of both RV longitudinal and strain may extend the use of dapagliflozin in patients with RVD but without signs and symptoms of RHF, representing the first drug for the prevention of the development of overt RHF. Additionally, current findings occur regardless LVEF, and are much more significative in patients with PH. (Graphical Abstract) the RV functional improvement is associated with significant reduction in NTproBNP levels, NYHA class, and adverse event rate. The reduction in NTproBNP levels demonstrated a direct effect in terms of decongestion and right heart function in patients treated with dapaglifozin. Previous studies showed the linear relationship between RV ejection fraction and natriuretic peptides increase as well as their relation with prognosis.^26^ These data arise from a large comprehensive echocardiographic study, although right heart catheterization (RHC) keeps the gold standard for PH and RA pressure calculation. However, RCH application in clinical practice is usually limited to patients with advanced HF, those with PH of uncertain causes, and subjects screened for heart transplantation.^16^ Interestingly, RV assessment by complete echocardiographic analysis can provide additional information on RV function pulmonary systolic pressure and RV-PA coupling.^27,28^

### Myocardial and haemodynamic effects of dapaglifozin

From a cellular and metabolic point of view, an animal model of chemically induced PH revealed that dapagliflozin treatment attenuated RV structural remodelling and improved RV function, through the restoration of the expression of Ca2 handling proteins and the promotion of cellular Ca2 uptake.^29^ An additional potential effect of Dapagliflozin is the capacity of attenuating collagen expression by increasing the production of matrix metalloproteinase 2 (MMP2) and matrix metalloproteinase 9 (MMP9) together with the reduction in oxidative stress, leading to the demonstration of a reduced fibrosis amount at RV level.^30^

Our results are in line with previous studies, demonstrating positive findings on haemodynamic parameters, BNP reduction, and congestion status-^31,32^ A post-hoc analysis of a small trial involving 37 HFpEF patients randomized to 24 weeks with dapagliflozin showed a reduction in pulmonary vascular load and an enhancement in dynamic RV-PA interaction during exercise.^33^ Considering the incremental prognostic value of TAPSE matched for PASP by echocardiographic examination, as reliable index of RV and PA elastance (Ees/Ea), current finding may provide an additive mechanism of SGLT2i treatment beneficial properties.^34–36^ The improvement of PASP/TAPSE observed in our data suggests that dapagliflozin treatment may have beneficial effects on pulmonary vascular resistance and viscoelastic forces leading to a better RV length-contractile forces relationship. Current haemodynamic changes may additionally reduce the ventricular-atrial gradient during systolic phase; indeed, the reduced afterload and the reduction in pulmonary resistance decrease RV enlargement driving towards a reduction in TR with global improvement in RV function and pressures.^37^

### Previous analyses

Due to its prognostic relevance and the lack of effectiveness of traditional therapy in influencing RV reverse remodelling, there is an unmet need to search for potential treatment capable of improving RV performance and possibly reducing ventricular pulmonary uncoupling. Despite various studies analysed the drug effects on LV remodelling and function, no significant data on RV function were reported: an exploratory analysis of Denmark registry showed Empagliflozin has a modest impact on LV remodelling and mass^38^ In a different single centre study, 6 months of dapagliflozin treatment improves LV and LA strain but, again, RV analysis was not described.^39^

Although current effects could be considered related to the class, contrasting data are currently published. Indeed, in a preliminary study with empagliflozin evaluating diabetic patients with coronary diseases, no significant effects on RV volumes mass and LVEF were observed between treated and control arm.^40^ Nevertheless, 12 weeks of Empagliflozin administration demonstrated relevant effects on wedge pressure and vascular resistance reduction without change in cardiac output.^41^ Notably, our findings are in line with the preliminary results deriving from the use of SGLT-2i in patients with systemic right ventricular dysfunction.^42,43^ Overall, to our knowledge, the present investigation represents the wider study investigated the effects of SGLT-2i on RV morphology and function, pulmonary arterial pressure, ventricular-arterial coupling, and TR in HF patients. Notably, it extends preliminary data deriving from small observational pilot studies, none of them including a complete and exhaustive evaluation focused on the RV.^44,45^ In addition, this is the first study investigating the separate effects of dapagliflozin on HF with regard to LVEF phenotypes and the presence of PH.

### Study limitations

Our study has some limitations, mainly related to the observational nature of the study. First, the observational, propensity-score–based design precludes causal inference, and residual confounding cannot be excluded. Second, RV function and PASP were assessed exclusively by echocardiography without invasive haemodynamic. Third, follow-up was limited to 6 months, so long-term durability of RV changes and impact on hard outcomes remain uncertain.

In addition, the gold standard for the comprehensive imaging evaluation of RV is cardiac magnetic resonance (CMR), which is able to evaluate RV in three-dimensional modality and to assess the whole RV chamber and parietal contractility. However, despite echocardiographic study encompassing some limitations related to the incomplete visualization of RV, it is the most available tool employed for right chamber evaluation.^46,47^ A similar argument could be discussed regarding the evaluation of PASP, mean pulmonary arterial pressure, RAP, RV elastance, and wedge pressure, with the RHC remaining the gold standard.^26^ Thus, haemodynamic and functional status could be better investigated by using both CMR and RHC. A more complete echocardiographic assessment should include the pulmonary artery pressure index (PAPI), however this parameter is not routinarely assessed by echocardiography and most of data comes from invasive studies. However, the echocardiographic study represents, to date, the most available and feasible instrument in the clinical practice. In addition, the RV maladaptation in HFpEF and HFrEF may subtend different pathophysiology with distinct mechanisms responsible for pulmonary hypertension and RV remodelling, and the present study does not identify specific mechanisms leading to the RV function improvement^48,49^; however, this would be out of the purpose of the present study.

## Conclusions

RV structural and functional alterations are the major prognostic determinants in HF, but any treatment demonstrated a positive impact on RV function and intracardiac pressure. Current comprehensive echocardiographic analysis offers a new potential mechanism of dapaglifozin in amelioration of RV mechanical and haemodynamic profiles. These echocardiographic changes support a potential mechanism of dapagliflozin involving reduced pulmonary vascular load, improved RV–PA coupling, and possibly direct effects on RV myocardial energetics and fibrosis, beyond LV reverse remodelling.

## Supplementary Material

xvag230_Supplementary_Data

## Declarations

### Data Availability

Data will be available upon specific Journal and reviewer request.

### Pre-registered Clinical Trial Number

None supplied.
