# Supplementary material for: Dapagliflozin effects on right ventricular function, pulmonary pressure and coupling: a propensity score analysis from the RIVED-HF
Source: ESC Heart Fail. 2026 Sep 1;13(5):xvag230. doi: 10.1093/eschf/xvag230 (PMC13639484; doi:10.1093/eschf/xvag230)
Supplement: Supplementary file 1 [file xvag230_supplementary_data.docx]

Supplementary Table 1. Differences in baseline characteristics among patients who were included or not in the study

|  | **Included patients (n=142)** | **Excluded patients (n=71)** | **p-value** |
| --- | --- | --- | --- |
| **Age (years)** | 78 (75- 84) | 77 (72-84) | 0.703 |
| **Gender male (%)** | 62 | 58 | 0.552 |
| **NTproBNP (pg/mL)**  **NYHA class 2 (%)**  **HYHA class 3 (%)** | 1031 (546.5-2000.5)  53  19 | 1046.5 (512.7- 1913)  50  13 | 0.738  0.628  0.245 |
| **LVEF (%)**  **Risk factors (%)**  Diabetes Mellitus  Hypertension  Dyslipidaemia | 45 (35- 53)  35  56  54 | 44 (36- 55)  44  48  49 | 0.976  0.231  0.243  0.497 |
| **Atrial fibrillation (%)**  **CKD (%)** | 36  30 | 30  39 | 0.357  0.149 |

SUPPLEMENTARY Figure 1 changes in NYHA class from baseline to 6 months follow-up in dapa and in control group

SUPPLEMENTARY Figure 2. Reasons for avoiding SGLT2Is treatment in control group
